# Polarized Micro-Raman Spectroscopy and 2D Convolutional Neural Network Applied to Structural Analysis and Discrimination of Breast Cancer

**DOI:** 10.3390/bios13010065

**Published:** 2022-12-30

**Authors:** Linwei Shang, Jinlan Tang, Jinjin Wu, Hui Shang, Xing Huang, Yilin Bao, Zhibing Xu, Huijie Wang, Jianhua Yin

**Affiliations:** 1 Department of Biomedical Engineering, Nanjing University of Aeronautics and Astronautics, Nanjing 210016, China; 2 Department of Pathology, Jiangsu Cancer Hospital, Jiangsu Institute of Cancer Research & the Affiliated Cancer Hospital of Nanjing Medical University, Nanjing 210016, China

**Keywords:** polarized micro-Raman spectroscopy, breast cancer, 2D-convolutional neural network, discrimination

## Abstract

Raman spectroscopy has been efficiently used to recognize breast cancer tissue by detecting the characteristic changes in tissue composition in cancerization. In addition to chemical composition, the change in bio-structure may be easily obtained via polarized micro-Raman spectroscopy, aiding in identifying the cancerization process and diagnosis. In this study, a polarized Raman spectral technique is employed to obtain rich structural features and, combined with deep learning technology, to achieve discrimination of breast cancer tissue. The results reconfirm that the orientation of collagen fibers changes from parallel to vertical during breast cancerization, and there are significant structural differences between cancerous and normal tissues, which is consistent with previous reports. Optical anisotropy of collagen fibers weakens in cancer tissue, which is closely related with the tumor’s progression. To distinguish breast cancer tissue, a discrimination model is established based on a two-dimensional convolutional neural network (2D-CNN), where the input is a matrix containing the Raman spectra acquired at a set of linear polarization angles varying from 0° to 360°. As a result, an average discrimination accuracy of 96.01% for test samples is achieved, better than that of the KNN classifier and 1D-CNN that are based on non-polarized Raman spectra. This study implies that polarized Raman spectroscopy combined with 2D-CNN can effectively detect changes in the structure and components of tissues, innovatively improving the identification and automatic diagnosis of breast cancer with label-free probing and analysis.

## 1. Introduction

According to the GLOBOCAN database released by the International Agency for Research on Cancer in 2020, female breast cancer was the most commonly diagnosed cancer worldwide and the most common cause of cancer death in women [1]. However, no drug has been developed to prevent breast cancer. Nowadays, breast cancer mortality can only be reduced by early diagnosis and treatment, which requires rapid and accurate detection.

The current gold standard for breast cancer screening is triple assessment using imaging (a combination of X-ray mammography and ultrasound), clinical examination, and histological assessment [2]. Mammograms generally provide 10–14% false positives and requires further testing. Ultrasound can reveal the shape, location and structure of tumors [3]. However, ultrasound has not enough specificity in distinguishing between benign and malignant tumors. Magnetic resonance imaging (MRI) can also be used to detect breast cancer and has a lower false positive rate at the middle and late stages; however, it is limited by high cost [4]. Once a tumor is suspected to be cancerous, the core biopsy procedure is performed. At present, the commonly used sampling methods mainly include needle core biopsy and surgical biopsy. Needle core biopsies will produce false negatives due to either small sampling volumes or sampling error, while surgical biopsies are an invasive method, causing pain and even complications for patients.

Raman spectroscopy is a powerful label-free technique with incredible potential for biological analysis; it can detect biochemical composition without any complex sample preparation and external reagents, and therefore, reduces the overall time consumption for rapid detection [5]. Moreover, in vivo and ex vivo analyses can be performed with special attachments such as an optical fiber Raman probe. Liu et al. [6] introduced the applications of Raman spectroscopy for the in vivo and ex vitro diagnosis of gastric cancer, and the methodology related to spectroscopy data analysis. In this sense, Raman spectroscopy can be applied for the early diagnosis of breast cancer [7]. Several groups have validated the feasibility of Raman microscopic spectroscopy in breast cancer diagnosis in form of tissue sections and have achieved valuable results [8,9,10]. However, the sectioning process increases the complexity of sample preparation, and the sectioning samples can hardly provide the complete biochemical information of the whole tissue. Microscopic Raman spectroscopy has been able to collect information concerning the composition and content changes of fatty acids and collagen, etc., in breast tissue [11,12]. Furthermore, Ragini et al. [13] discussed significant advancements in the use of laser Raman spectroscopy in surgical breast cancer diagnosis, with an emphasis on statistical and machine learning strategies employed for precise, transparent and real-time analysis of Raman spectra. However, the cancerization information concerning the structural organization of the main components in breast tissue, which might be involved in cancer invasion and metastasis, is hard to acquire.

Polarized micro-Raman spectra can provide meaningful information about the conformation and orientation of the tested biomolecule. Breast tissue consists of adipose tissue and connective tissue. The distribution of collagen fibers, the main component of connective tissue, is often optically anisotropic. This property makes compositional and structural differences of collagen fibers likely to become significant under polarization conditions. Ly et al. [14] applied polarized microscopic Raman spectroscopy to study basal cell carcinoma, finding the enhanced spectral changes between tumors and healthy tissues and the related structural changes. Daniel et al. [15] used polarized Raman spectroscopy for cervical cancerous tissue detection, which achieved a better recognition effect and obtained additional information about tyrosine, collagen and DNA orientation in the polarization spectra of cancerous tissue. Lin et al. [16] developed a more powerful blood analysis method based on polarized surface enhanced Raman spectroscopy technology for non-invasive and sensitive colorectal cancer (CRC) detection. Abramczyk et al. [17] used polarized Raman spectroscopy and imaging technology to characterize the isotropic and anisotropic vibrational responses in noncancerous and cancerous human breast tissues. The results revealed that polarized Raman spectroscopy has better diagnostic potential than conventional Raman spectroscopy. Based on the above publications, this research will be more valuable by adding deep learning to it for cancer diagnosis.

Introducing deep learning into Raman spectroscopy can make spectral analysis more effective and automatic. A convolutional neural network (CNN) is a representative structure of the deep learning model and is currently a research focus in deep learning. It has a strong capability for extracting higher-level features. A platform for the Raman signature discrimination of extracellular vesicles based on CNN was suggested by Lee’s group, which can identify prostate cancer with no less than 93% accuracy [18]. Yan proposed an ensemble CNN framework to distinguish tongue squamous cell carcinoma from non-tumor tissue and obtained a high discrimination accuracy of 99.2% [19].

This paper will experimentally demonstrate that polarized Raman spectroscopy has greater potential for breast cancer research and identification than conventional Raman spectroscopy. A spectral analysis and T-test for band intensity will be performed to evaluate the bio-structural changes and statistical significance of spectral differences between normal and cancerous tissues by conventional and polarized Raman spectroscopy. A two-dimensional CNN (2D-CNN) model based on the polarization Raman spectral image will be constructed to identify breast cancer tissue.

## 2. Materials and Methods

### 2.1. Sample Preparation

The breast tissues were harvested from Jiangsu cancer hospital patients, which was approved by the local Ethics Committee after informed consent. The samples were taken into the lab at −5 °C for 1 h after being extracted from their biological surroundings and cleaned with normal saline. Then, they were detected directly with our lab-made Raman spectrometer without any sample preparation. The operations were selected to make our results and clinical outcome as consistent as possible. The breast tissue samples were collected from 20 patients. All patients provided cancerous tissues, 10 of the patients provided normal tissues, 12 of the patients provided paracancer tissues (20 cancerous samples, 10 normal samples and 12 paracancer samples in total). The patients’ ages were between 27 and 68 years and they were at different stages of the disease. The samples were identified in clinic and taken from different positions with specific distances. The size of the samples were about 0.3 cm × 0.3 cm × 1 cm.

### 2.2. Spectral Acquisition

A lab-made micro-Raman spectroscopy system was used in the experiment [20]. It is composed of a 785 nm laser light source (IPS), a cooling CCD detector (Andor), an external optical path system, a microscopic system, a dispersion system and a computer processing and display system. The dispersion system contains a Holo blazed grating (Newport, 53006BK01-230H, 1200 G 800 nm Holo) and a slit of 50 μm. The spectral range is 500~2000 cm^−1^ with a resolution of 3 cm^−1^. During spectral detection, the laser light excitation light (50 mW) entered the microscope system and was focused on the sample in a circular area of 20 μm diameter through the 20× objective lens, the excitation time was 30 s to not burn the samples. Subsequently, the Raman scattering was collected by the collection system into the CCD and converted into the required spectrum by the computer processing system. The sample was moved for spectral collection with a ca. 2 mm interval on the stage each time to obtain the statistical results.

The polarization angle of the incident light was adjusted by a half-wave plate installed at the entrance of the microscope, and then this was rotated to change the polarization angle of the incident light while keeping the excitation power constant. The polarized spectra were detected at every 30° from 0° to 360° at the points where the conventional spectra were detected. All detection conditions remain consistent with the conventional spectra detection. In total, 10 points were detected for each sample with the same space interval.

### 2.3. Spectral Preprocessing

After the fluorescence background was automatically removed by Vancouver Raman algorithm software [21], the normalized mean spectra were obtained by Origin Pro 2017. By using the line connecting the adjacent wave trough of each peak as the baseline, the integral area of characteristic bands with significant changes was calculated by Python. IBM SPSS 22 was used to conduct an independent sample T test for spectral band intensity (band area) to evaluate the statistical significance of spectral differences between normal and cancerous tissues by conventional and polarized spectroscopy (*p* < 0.05).

### 2.4. Data Augmentation and Data Set

**Figure 1:**
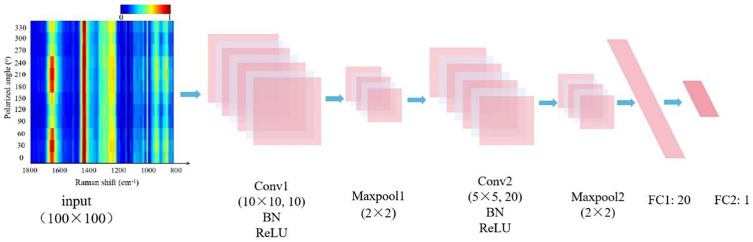
The architecture of the 2D-CNN discriminant model. Two convolutional layers were abbreviated as Conv1 and Conv2, as well as two fully connected layers as FC1 and FC2. The max-pooling layers are Maxpool1 and Maxpool2, respectively.

The 12 polarized spectra of each detection point were arranged into a 12 × 1780 matrix from 0 to 360°, and a 100 × 100 pseudo-color image was obtained after density slicing, scaling and clipping (the input image in Figure 1). In this study, 300 pseudo-color image data were finally obtained, including 100 from normal samples and 200 from cancerous samples.

In order to fully evaluate the CNN model’s stability and the over-fitting risk, a k-fold cross validation (k = 10) was performed to divide the training data and the test data. Specifically, the pseudo-color image data were divided into 10 groups according to the number of patients. Each group contained two cancerous samples and one normal sample, and was selected as the test set in turn, while the rest of the groups were used for training. In this way, the training was repeated 10 times, and all image data could be fully evaluated.

The number of cancerous samples was not balanced with that of normal samples, which might affect discrimination performance. To solve this problem, in each cross validation, three simple and effective data augmentation methods were used to expand the amount of training data without introducing additional marking costs, which were (1) Flip in horizontal and vertical; (2) Rotation: to rotate 90°, 180° and 270°, respectively; (3) Noise injection: Gaussian noises with mean values of 0.1, 0.2 and 0.3 were added, respectively. After that, the training data of cancerous and normal samples were expanded to 5000, simultaneously. Then, all the expanded data were divided into a training set and a validation set in a ratio of 7:3.

### 2.5. The 2D-CNN Model Building and Training

A 2D-CNN model consisting of an input layer, two convolutional layers (Conv1, Conv2), two fully connected layers (FC1, FC2) and two max-pooling layers (Maxpool1, Maxpool2) was constructed by Python. The architecture of the model is shown in Figure 1. All input images were derived from pseudo-color images of 12 polarized Raman spectra at each detection point, and then input in Conv1, which extracts features from the input data via convolution operations. Each convolution layer was followed by a BN layer to improve the generalization capability of the feedforward neural networks. After processing, the output features would be close to the standard normal distribution and then activated by the Leaky Rectified Linear Unit (ReLU) function. The (maximum) pooling layer was used for dimensionality reduction of the data from the convolutional layer and decreasing overfitting. The pooling kernel size was 2 × 2, and the stride size was 2. The fully connected layer made use of the results of the convolution and pooling processes to classify the image into a label (cancerous and normal samples were labeled as 0 and 1, respectively).

A backpropagation algorithm was used to update the weight of the network model. Binary Cross-Entropy was used as the loss function. Batch normalization (BN) layers, dropout layers, and an Adam optimizer were introduced to optimize the network model. The BN method was adopted to improve the training speed and reduce overfitting. A portion of the neurons in iteration were temporarily inactivated by dropout layers, and the risk of overfitting was decreased.

In order to evaluate the performance of the 2D-CNN model, a K nearest neighbor (KNN) classifier was also constructed by Python to learn and predict the above samples. During training, the KNN, settings of the cross validation, and the division of the datasets were consistent with that of the 2D-CNN. Moreover, a CNN model based on the conventional Raman spectra was established for further comparison with the 2D-CNN model. Since the conventional Raman spectrum is a one-dimensional vector, its discrimination model was established using one-dimensional CNN (1D-CNN) in this study. The loss function, optimizer, and network depth of 1D-CNN were consistent with 2D-CNN.

## 3. Results

### 3.1. Raman Spectral Analysis

**Figure 2:**
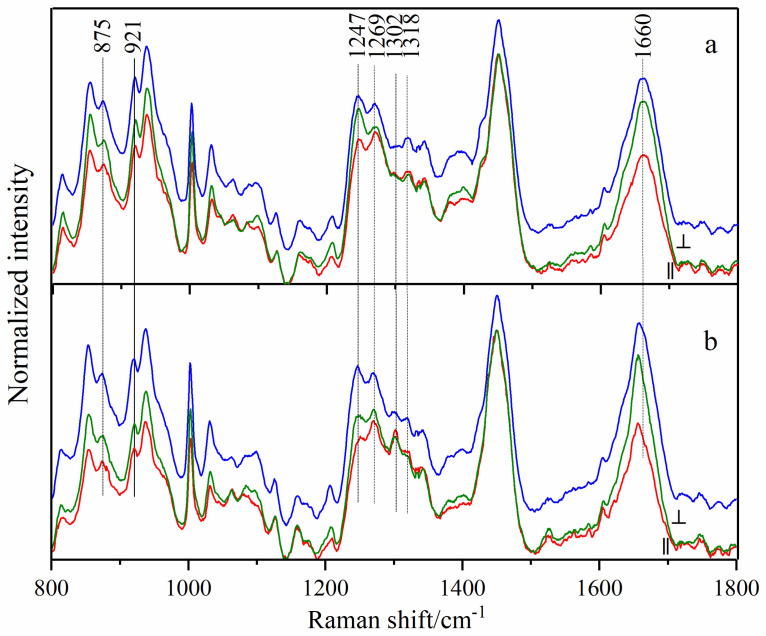
Polarized (red lines for parallel and green lines for perpendicular) and conventional (blue lines) Raman spectra of protein collected from normal (**a**) and cancerous (**b**) tissues. All shown are average spectra, which were normalized to the band of 1450 cm^−1^ to reduce the influence of tissue heterogeneity, respectively.

**Figure 3:**
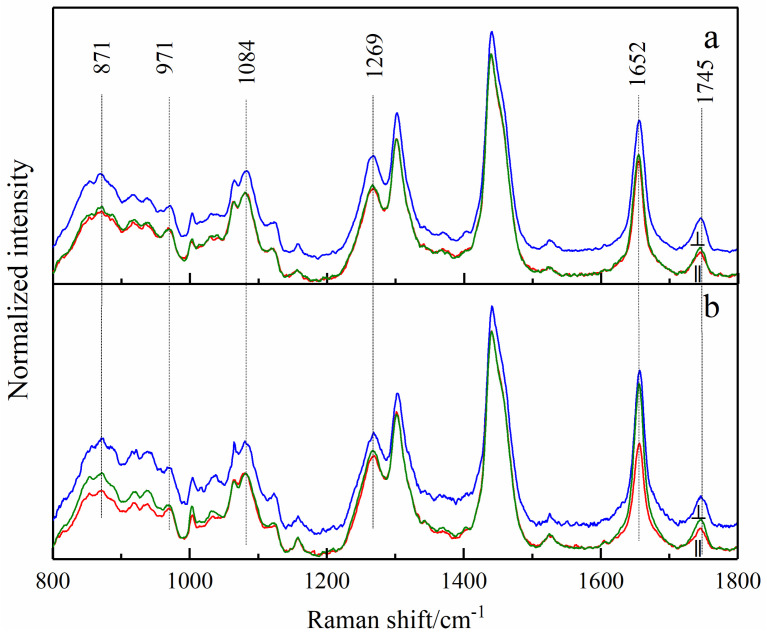
Polarized (red lines for parallel and green lines for perpendicular) and conventional (blue lines) Raman spectra of lipids collected from normal (**a**) and cancerous (**b**) tissues. All those shown are average Raman spectra, which were normalized to the band of 1442 cm^−1^ to reduce the influence of tissue heterogeneity, respectively.

There are heterogeneous structures in breast tissue [12], which shows the separation of protein and lipid to some extent. Therefore, the collected Raman spectra can be divided into protein spectra and lipid spectra, as shown in Figure 2 and Figure 3, respectively. There are significant differences between the spectra of the two components. The amide III (1247 cm^−1^) and phenylalanine (1003 cm^−1^) bands only exist in the protein spectra as strong bands [22,23]. The bands at 1084 cm^−1^ and 1745 cm^−1^, corresponding to C-O-C stretching vibration and C=O stretching vibration, obviously exist in the lipid spectra [24]. It must be noted that the bands at the same wavenumber have different attributions since they originate from different components; therefore, the Raman spectra of proteins and lipids are separately studied in this paper.

Figure 2 shows the parallel (red lines) and perpendicular (green lines) polarized Raman spectra and conventional Raman spectra (blue) of protein collected from normal (a) and cancerous (b) tissues. All those shown are average spectra, which were normalized to the band of 1450 cm^−1^ to reduce the influence of tissue heterogeneity, respectively. The Raman band assignments of protein spectra are listed in Table 1. The significant differences between normal and cancer tissues are mentioned below.

The amide I band at 1660 cm^−1^ in the normal samples shifted to 1656 cm^−1^ after cancerization, which was present in both conventional and polarized spectra. The amide III band is a characteristic collagen band composed of two peaks at 1247 cm^−1^ and 1269 cm^−1^. Although the two peaks had no significant difference between cancerous and normal tissues in conventional Raman spectra, they varied with the change of polarization angle in polarized Raman spectra. The intensity of the 1247 cm^−1^ peak in the perpendicular polarized spectra of normal tissue exceeded that of 1269 cm^−1^; the opposite was true in parallel polarized spectra. However, in cancerous tissues, the intensity of the 1247 cm^−1^ peak in either parallel or perpendicular polarized spectra was lower than that of 1269 cm^−1^. This phenomenon indicates that the collagen structure changes after cancerization, and optical anisotropy weakens.

In addition, the two bands of the twisting vibration of CH_2_ in collagen showed an opposite trend in normal and cancerous tissues; that is, the spectral intensity of the 1318 cm^−1^ band in the normal tissue spectra was stronger than that of 1302 cm^−1^ band, while it became weaker than the latter in the cancerous case. This change was more obvious in polarized spectra, which might be related to the change in the orientation of CH_2_ groups in the protein.

Table 2 shows the difference analysis of Raman intensity between normal and cancerous breast tissues under the conditions of conventional and polarized spectra (only parallel polarized spectra were considered), respectively. The bands of proline (921 cm^−1^), phenylalanine (1032 cm^−1^), and collagen (1302 cm^−1^) had no obvious difference in conventional Raman spectra (*p* = 0.113, 0.960, 0.063), but showed a significant difference in polarized spectra (*p* = 0.008, 0.034, 0.035). In conclusion, polarized spectra could significantly magnify the anisotropy differences between normal and cancerous tissues, resulting in significant statistical differences in the intensity of the corresponding feature bands.

Furthermore, the band areas with standard deviation of the above protein peaks are listed in Table 3. For the bands of 921, 1032 and 1302 cm^−1^, the differences of band area between cancerous and normal tissues were more obvious in polarized spectra, which was consistent with the above results, and proved the effectiveness of polarized Raman spectroscopy in the diagnosis of protein spectra of cancerous breast tissues. The band areas of 921, 1032 and 1302 cm^−1^ in polarized Raman spectra may become the key for breast cancer diagnosis.

Figure 3 shows the conventional and polarized spectra of the lipids, and Table 4 shows their Raman band assignments. Due to the sample differences among different patients, normalization can make the spectra display better. The band at 1442 cm^−1^ in the lipid spectra was attributed to the deformation vibration of CH_2_. Therefore, the normalized average spectra can represent the intensity variation of other bands relative to 1442 cm^−1^.

The Raman intensity at the 1652 cm^−1^ band (C=C stretching) of cancerous tissue was significantly higher than that of normal tissue, suggesting that the intensity ratio (I_1652_/I_1442_) of breast tissue increased after cancerization [27]. The value of I_1652_/I_1442_ could be used to characterize the ratio of the number of C=C groups to the number of CH_2_ groups in breast tissues ($N_{C=C}/N_{CH_{2}}$) [30], whose increase indicated that the synthesis of unsaturated fatty acids in breast tissues increased and saturated fatty acids relatively decreased during cancerization. The relative changes between saturated and unsaturated fatty acids affected the fluidity and viscosity of the cell membranes, and further affected the diffusion and rotation of proteins and other biomolecules within the membrane [31].

The band at 1269 cm^−1^ (=C-H deformation vibration of lipid) in the conventional Raman spectrum showed no significant difference between normal and cancerous tissues. However, its intensity in cancerous cases was increased in polarized spectra. This indicated that the content of unsaturated fatty acids was increased in cancerous tissues, which was consistent with the analysis results above.

As with the collagen spectra, a difference analysis for the bands that showed apparent differences in lipid spectra was performed, as shown in Table 5. The results showed that the bands at 871, 971, 1084 and 1652 cm^−1^ did not show significant difference in conventional Raman spectra (*p* = 0.767, 0.539, 0.781, 0.077), but showed an obvious difference in polarized Raman spectra (*p* = 0.005, 0.011, 0.043, 0.033).

The band areas with standard deviation of the above lipid peaks are listed in Table 6. For the band at 1745 cm^−1^, the band areas of polarized and conventional Raman spectra both showed an increase in cancerous tissues. However, for the bands at 871, 971, 1084 and 1652 cm^−1^, the differences of band area between cancerous and normal tissues were more obvious in polarized spectra. These results further confirmed the advantages of polarized Raman spectroscopy in the diagnosis of breast cancer compared with conventional Raman spectroscopy.

Although many differences were found in the Raman spectra of cancerous and normal tissues, such subtle changes in vibration modes may not be sufficient for cancer diagnosis, yet. Thus, CNN was introduced to extract the shallow features in Raman spectra into abstract deep features, and automatically find out the major and subtle spectral differences between cancerous and normal tissues.

### 3.2. The 2D-CNN Discrimination Analysis

In this study, a small learning rate (learning rate = 5 × 10^−5^) was set to improve the convergence results. In addition, to realize the visualization of the training effect, the model introduced the Visdom server, a professional drawing plugin based on the PyTorch framework, which was instructed to display the loss function and the accuracy curves of the 2D-CNN. The training was stopped when the loss function curves no longer dropped significantly to avoid overfitting.

**Figure 4:**
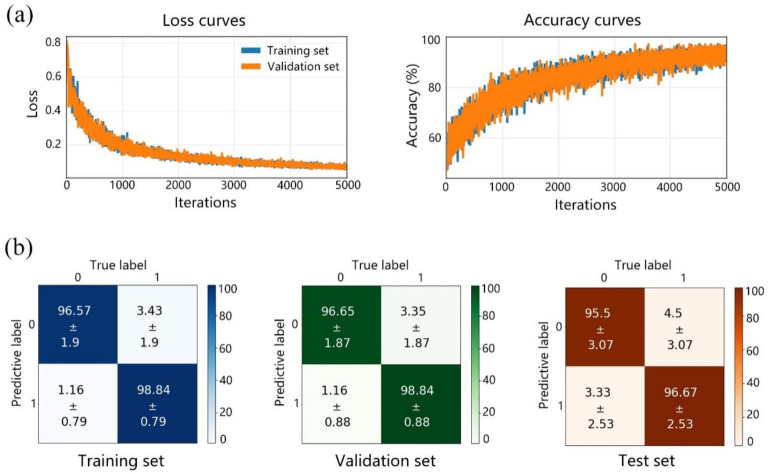
(**a**) The loss function curves and accuracy curves, and (**b**) confusion matrices of the training set, validation set, and test set in the 2D-CNN prediction. 0: cancerous, 1: normal; unit: % in horizontal and vertical axes of (**b**).

The curves of the loss function and training accuracy are shown in Figure 4a. The abscissas indicate the number of iterations in training. The ordinates express the loss value and the overall accuracy, respectively. The loss function curves showed a downward trend, and both the training and validation sets eventually reached a stable value close to 0, indicating that the training of the CNN model was effective and stable. Similarly, the accuracy curves trended to stabilize after 5000 iterations.

After training, the training set, validation set, and test set were predicted by the 2D-CNN. The results and their confusion matrices are shown in Figure 4b. Each item in the matrices is the average predictive ratio with its respective mean squared error (MSE) of 10 times cross validation. The values on the diagonal squares indicate successful prediction (accuracy), while the other values in the remaining squares indicate the possibility of miss-predictive results (the lower left value represents a false negative, and upper right value represents a false positive). The total accuracies of the training set, validation set and test set were 97.71%, 97.75% and 96.01%, respectively, which were the average values of the diagonal squares in the matrices.

The trained 2D-CNN model obtained higher accuracies for the normal sample, which might be caused by its smaller data amount. However, the relatively close accuracies of the training set, validation set and test set with small MSE values indicate excellent stability of the 2D-CNN model and small risk of overfitting.

**Figure 5:**
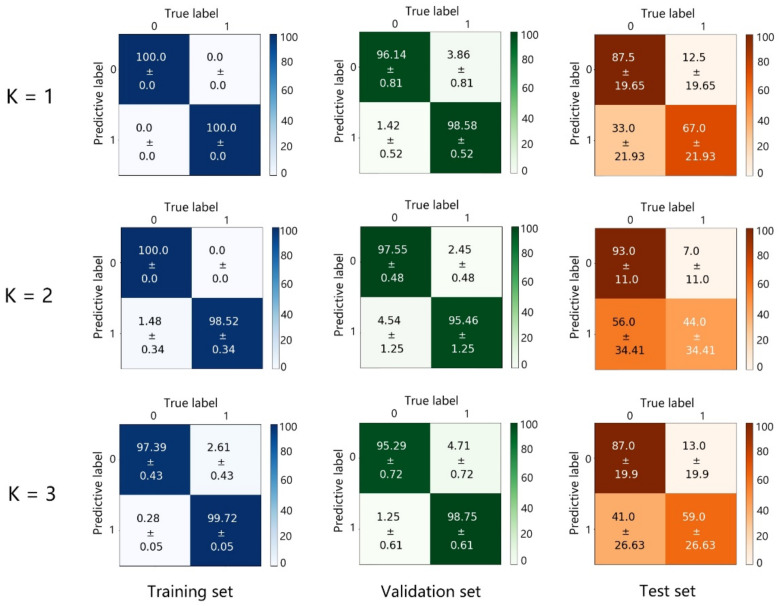
Confusion matrices of the training set, validation set, and test set predicted by the KNN classifier under different K values. 0: cancerous, 1: normal; unit: % in horizontal and vertical axes.

Next, the KNN classifier was used to learn and predict the above data. Figure 5 shows the predictive results of the training set, validation set, and test set under different K values. Although the KNN classifier achieved higher accuracies for the training set and validation set than the 2D-CNN under different K values, the accuracies of the test set were very low and unstable, which reveals the severe overfitting and poor prediction ability of the KNN. These results demonstrate the advantages of the 2D-CNN.

The results of the two models (2D-CNN and 1D-CNN) combined with polarized and conventional Raman spectroscopy, respectively, are shown in Table 7. The accuracies of the 2D-CNN model are 97.71%, 97.75% and 96.01% for the training set, validation set, and test set, respectively, which are clearly higher than those of the 1D-CNN model, at 92.0%, 92.8%, and 92.0%. This suggests that the discrimination performance of the 2D-CNN model is significantly better than that of the 1D-CNN model; further, the polarized Raman spectroscopy has a more excellent discriminant ability.

## 4. Discussion

This study shows the potential interest of polarized Raman spectroscopy in analyzing the structural changes of collagen fibers in breast cancer tissues and normal tissues. According to the compositional heterogeneity of breast tissue, the spectra were divided into two categories of proteins and lipids for analysis. No matter which one was investigated, the difference analysis showed that polarized spectra can significantly enlarge the spectral difference between cancerous tissue and normal tissue, indicating that PMRS can achieve better spectral discrimination. The band difference of amide I is reduced in polarized spectra due to the band of amide I containing a variety of protein secondary structure information [32], and different secondary structures show different polarization characteristics. Although the band difference of amide I does not increase, it still retained significant differences (*p* = 0.019) in the polarized Raman spectra.

Some information about the orientation change of collagen fibers was also found in polarized Raman spectra. The significant differences of the collagen amide III band existed in the polarized spectra of normal and cancerous tissues. The band of amide III is a characteristic band of collagen, with a unique bimodal structure of 1247 cm^−1^ and 1269 cm^−1^. In view of this feature, this study compared the bimodal ratio (I_1247_/I_1269_) between the spectra of normal and cancerous tissues. To further study the cancerization mechanism, polarized Raman spectra of paracancerous tissues (belonging to the undiseased tissues and being ca. 3 cm away from cancerous tissues) were also detected and analyzed together with normal tissues and cancerous tissues.

**Figure 6:**
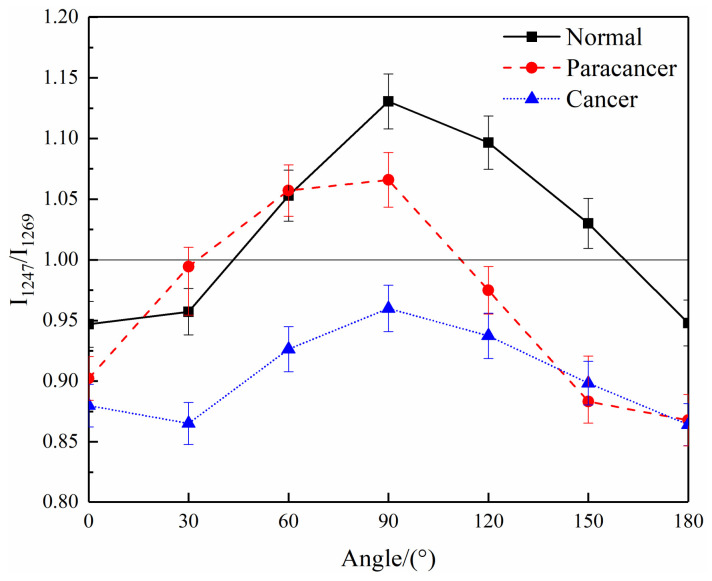
The trends of the bimodal ratio (I_1247_/I_1269_) in the spectra of cancerous, paracancerous and normal breast tissues with the polarization angle.

Figure 6 shows the trends of the I_1247_/I_1269_ values of the polarized Raman spectra of normal, paracancerous and cancerous tissues with the polarization angle. The bimodal ratios in normal and paracancerous tissues is less than 1 at 0° and over 1 at 90°. In contrast, the bimodal ratio of cancerous tissues never exceeds 1, which suggests that the amide III in cancerous tissues does not show prominent polarization characteristics in the same way as normal tissues. The bimodal ratio in cancerous cases is lower than normal and paracancerous tissues, whether it is 0° or 90°. Some researchers attributed the band at 1247 cm^−1^ to the 3_10_ helix of collagen and the band at 1269 cm^−1^ to the α helix [33,34,35]; and it is considered that the above changes are caused by the decrease of the 3_10_ helix in collagen. The bimodal ratio of amide III in collagen spectra is also related to its orientation [36]. The trend of the I_1247_/I_1269_ value of the Raman spectra of normal tissues is unique to horizontal collagen fibers, while it is not reflected in cancerous tissues [36]. This suggests that the orientation of collagen fibers changes in the process of cancerization. When cancerization occurs in breast tissue, the orientation of collagen fibers in the tissue gradually changes from the original horizontal orientation to the vertical orientation, which is more conducive to the spread of cancerous cells [37]. Therefore, a mixture of horizontal and vertical collagen fibers will appear in the cancerous tissue and lead to the ratio result in Figure 6. The reduction of the I_1247_/I_1269_ ratio of cancerous tissue at all angles may become the key for cancer diagnosis.

Although the spectra of paracancerous tissues, which are very close to cancerous tissues, do not show the spectral characteristics of cancerous tissues, the bimodal ratio is lower than that of normal tissues. These paracancerous characteristics are imperceptible and hard to be detected by other imaging techniques [36]. However, the polarized Raman spectra is sensitive to the kinds of subtle changes happening at every moment.

Theoretically, collagen exists in the form of collagen fibers, and its secondary structure is a triple helix composed of three strands of polypeptide chain [38]. This structure will change due to the hydroxylation of some amino acids (such as proline), that will affect the hydrogen bond mode and the secondary structure of collagen [38]. Therefore, the relative content changes of hydroxyproline (875 cm^−1^) and proline (921 cm^−1^) between cancerous and normal tissues were analyzed to investigate the reasons for the changes in the orientation of the collagen fibers. Table 8 shows the intensity of the integral area ratios (I_875_/I_921_) of proline and hydroxyproline bands in the average spectra of the cancerous and normal tissues. It was found that whether using conventional or polarized Raman spectra, the content of hydroxyproline increased, relatively, after cancerization. In this sense, the hydroxylation degree of collagen became higher after cancerization, resulting in the changes of the hydrogen bond mode of collagen fibers. Consequently, the changes in the structure and orientation of the collagen fibers and a redshift of the amide I band were induced simultaneously.

In previous studies [11,12,39], the detailed structural changes during breast cancerization have been characterized and discussed by conventional Raman spectroscopy, autofluorescence imaging and polarization imaging technology. On this basis, this study focused on exploring the application of polarized Raman spectroscopy in breast cancer diagnosis, and the possibility of combining polarized Raman spectroscopy with 2D-CNN.

In the training of the 2D-CNN, a key issue was to evaluate and reduce the risk of overfitting. Thus, the following methods were performed in this study: (1) K-fold cross validation was used for the full evaluation of all data. (2) Three data augmentation methods were introduced to expand the data amount and balance the size of cancerous and normal samples. (3) BN layers and dropout layers were added into the 2D-CNN model to overcome the overfitting. (4) The training was stopped when the loss function curves no longer dropped, which is known as the ‘early stop strategy’. (5) A KNN classifier was introduced to process the same data set for comparison. The final results confirmed the effectiveness of the above methods.

The deep learning discriminant models, 2D-CNN and 1D-CNN, were established based on polarized and conventional Raman spectra, respectively. The 2D-CNN model based on polarized Raman spectroscopy showed a superior capacity for distinguishing the cancerous tissues from normal tissues from the latter. Some spectra from normal samples in the 2D-CNN test set were identified as cancerous ones, which is ascribed to the fact that the normal tissues used in the experiments were not taken from perfectly healthy people but donated from cancer-free sites in the breast cancer patients. The tissue microenvironment had, probably, changed before it became cancerous; with the components and bio-structure subtly altered in the normal tissue, as explained in the discussion above.

Compared with normal Raman spectra, polarized Raman spectra can provide more orientation information, which was found to have the potential for cancerous breast tissue diagnosis. More importantly, the Raman spectra acquired at a set of linear polarization angles varying from 0° to 360°, which were combined into a two-dimensional matrix in parallel. In this situation, 2D-CNN not only extracted the respective characteristics of the spectra, but also learned the spectral changes under different polarization angles, which took the orientation changes of breast tissues into account. Therefore, the polarized Raman spectra based 2D-CNN achieved better results than those of normal Raman spectra based 1D-CNN. Polarizing was of great importance to obtain the results of this study.

The purpose of this study was to explore a rapid and label-free diagnostic method for breast cancer. Therefore, Raman spectra were obtained directly from the breast tissue. In future, biosensing applications or a surface enhanced Raman spectroscopy method can be introduced into the tissue section samples for better Raman spectra and to further improve the 2D-CNN results.

## 5. Conclusions

Polarized Raman spectroscopy was applied in the research on breast cancer for the first time. It discovered much more biochemical information and more significant differences between cancerous and normal tissues than conventional Raman spectroscopy. The polarized Raman spectral analysis of breast tissues discloses the structural changes in collagen orientation, and suggests that the orientation of collagen fibers gradually transforms during cancerization, as shown in the further study on paracancerous tissues. The results above are very helpful for the study of the metastasis and prognosis of cancerous cells. The discriminant model of 2D-CNN based on polarized Raman spectral images verifies that it has an excellent capacity to distinguish between cancerous tissues and normal tissues. This technique yields a higher accuracy when compared with the conventional Raman spectroscopic technique. Polarized Raman spectroscopy with deep learning will be a valuable and innovative technique for cancer probing, and has the potential to be developed as a sensitive and label-free optical tool for assessing aggressiveness and invasion during tissue cancerization.

## Figures and Tables

**Table 1:** Raman band assignments of protein in normal and cancerous breast tissues [22,23,24,25,26,27,28,29].

Raman Shift (cm^−1^) of Normal (Cancerous) Tissues	Mode of Vibration	Assignment	Spectral Difference and Cancer vs. Normal Breast
875	ν(C—C)	Hydroxyproline in collagen	Decrease, more obvious in polarized spectra
921	ν(C—C)	Proline in collagen	Decrease, more obvious in polarized spectra
1003	ν(C—C)	Phenylalanine	Decrease
1032	δ(CH_2_CH_3_)	Phenylalanine in collagen	Decrease
1247	δ(N—H)	Amide III	Increase
1269	ν(C—N)	Amide III	\
1302	γ_t_(CH_2_)	Collagen	Increase, more obvious in polarized spectra
1318	γ_t_(CH_2_)	Collagen	Decrease, more obvious in polarized spectra
1450	δ(CH_2_, CH_3_)	Proteins	Decrease
1660 (1656)	ν(C=O)	Amide I, α-helix	Red shift, increase, more obvious in conventional spectra

Note: ν-stretching coordinate; δ-deformation; γ_t_-twisting coordinate.

**Table 2:** Statistical differences (*p*-values) of protein band intensities between normal and cancerous breast tissues collected with conventional and parallel PMRS, respectively.

Method	Raman Band (cm^−1^)									
	875	921	1003	1032	1247	1269	1302	1318	1450	1660
Polarized	0.019	0.008	0.026	0.034	0.562	0.397	0.035	0.067	0.319	0.072
Conventional	0.023	0.113	0.025	0.96	0.681	0.685	0.063	0.167	0.271	0.02

Note: *p* < 0.05 was considered statistically significant.

**Table 3:** Band area with standard deviation of each protein Raman peak.

		Raman Band (cm^−1^)									
		875	921	1003	1032	1247	1269	1302	1318	1450	1660
Cancerous Polarized	Area	1.03	1.17	4.16	2.66	5.88	1.64	0.37	0.18	40.82	49.99
	Std	0.64	0.54	0.85	0.87	1.64	0.86	0.57	0.28	3.94	9.23
Normal Polarized	Area	1.22	1.46	4.33	2.72	5.76	1.72	0.13	0.27	43.94	48.21
	Std	0.62	0.5	0.79	0.78	1.56	0.86	0.55	0.31	4.38	8.26
Cancerous Conventional	Area	1.09	1.34	4.78	2.73	6.42	1.42	0.32	0.24	39.07	56.52
	Std	0.63	0.51	1.28	0.77	1.53	0.7	0.68	0.26	5.38	7.43
Normal Conventional	Area	1.15	1.49	4.9	2.82	6.24	1.52	0.25	0.29	42.2	48.68
	Std	0.49	0.53	1.31	0.94	2.18	0.97	0.95	0.34	5.61	8.94

**Table 4:** Raman band assignments of lipids in normal and cancerous breast tissues [22,23,24,25,26,27,28,29].

Raman Shift (cm^−1^) of Normal Tissues	Mode of Vibration	Assignment	Spectral Difference and Cancer vs. Normal Breast
871	ν(N^+^(CH_3_)_3_)	Phospholipids	Decrease, more obvious in polarized spectra
971	ν(C—C)	Phospholipids	Decrease, more obvious in polarized spectra
1032	δ(CH_2_CH_3_)	Phospholipids	Decrease
1084	ν(C—O—C)	Phospholipids	Decrease, more obvious in polarized spectra
1269	ν(PO_2_), δ(=C—H)	Lipids	\
1302	δ(=C—H)	Lipids	\
1442	δ(CH_2_)	Lipids	\
1652	ν(C=C)	Unsaturated bonds of lipids	Increase
1745	ν(C=O)	Lipids	Increase

Note: ν-stretching coordinate; δ-deformation; γ_t_-twisting coordinate.

**Table 5:** Statistical differences (*p*-values) between lipid band intensities of normal and cancerous breast tissues collected with conventional and parallel polarized spectra, respectively.

Method	Raman Band (cm^−1^)								
	871	971	1032	1084	1269	1302	1442	1652	1745
Polarized	0.005	0.011	0.052	0.043	0.68	0.375	0.214	0.033	0.008
Conventional	0.767	0.539	0.852	0.781	0.726	0.454	0.139	0.077	0.045

Note: *p* < 0.05 was considered statistically significant.

**Table 6:** Band area with standard deviation of each lipid Raman peak.

		Raman Band (cm^−1^)								
		871	971	1032	1084	1269	1302	1442	1652	1745
Cancerous Polarized	Area	0.24	1.06	1.18	1.36	6.94	8.47	37.55	17.54	2.98
	Std	0.28	1.07	0.76	0.83	3.41	2.30	5.74	4.38	1.09
Normal Polarized	Area	0.43	1.49	1.31	1.72	6.81	8.68	36.89	14.21	1.89
	Std	0.63	1.13	1.2	2.45	4.71	4.49	8.64	4.59	1.76
Cancerous Conventional	Area	0.63	1.23	0.76	2.12	7.89	8.72	38.91	19.93	3.98
	Std	0.30	0.58	0.96	0.63	3.82	1.70	1.80	1.83	0.98
Normal Conventional	Area	0.64	1.31	0.83	2.25	8.08	8.52	37.33	18.43	3.15
	Std	0.46	0.63	0.50	0.35	1.43	1.22	2.77	0.93	0.46

**Table 7:** The discrimination results of normal and cancerous breast tissues by PMRS image with 2D-CNN and conventional Raman spectroscopy with 1D-CNN.

Algorithm	Training Set	Validation Set	Test Set
2D-CNN	97.71%	97.75%	96.01%
1D-CNN	92.0%	92.8%	92.0%

**Table 8:** Characteristic intensity of the integral area ratios of proline and hydroxyproline in cancerous and normal tissues.

	Ratios (I_875_/I_921_)	
	Conventional	Polarized
Cancerous	0.79 ± 0.15	0.81 ± 0.22
Normal	0.74 ± 0.09	0.76 ± 0.14

## Data Availability

The data presented in this study are available on request from the corresponding author.
